# Fabrication and optimization of 3D printed gelatin methacryloyl microneedle arrays based on vat photopolymerization

**DOI:** 10.3389/fbioe.2023.1157541

**Published:** 2023-04-28

**Authors:** Dilruba Baykara, Tuba Bedir, Elif Ilhan, Mehmet Eren Mutlu, Oguzhan Gunduz, Roger Narayan, Cem Bulent Ustundag

**Affiliations:** ^1^ Center for Nanotechnology and Biomaterials Application and Research (NBUAM), Marmara University, Istanbul, Turkey; ^2^ Department of Bioengineering, Faculty of Chemical and Metallurgical Engineering, Yildiz Technical University, Istanbul, Turkey; ^3^ Department of Metallurgical and Materials Engineering, Faculty of Technology, Marmara University, Istanbul, Turkey; ^4^ Department of Bioengineering, Faculty of Engineering, Marmara University, Istanbul, Turkey; ^5^ Department of Metallurgical and Materials Engineering, Faculty of Chemical and Metallurgical Engineering, Yildiz Technical University, Istanbul, Turkey; ^6^ Health Biotechnology Joint Research and Application Center of Excellence, Istanbul, Turkey; ^7^ Joint Department of Biomedical Engineering, University of North Carolina, Chapel Hill, NC, United States

**Keywords:** gelatin methacryloyl, hydrogel, microneedle, vat photopolymerization and additive manufacturing, 3D printing

## Abstract

Microneedles (MNs) are micrometer-sized arrays that can penetrate the skin in a minimally invasive manner; these devices offer tremendous potential for the transdermal delivery of therapeutic molecules. Although there are many conventional techniques for manufacturing MNs, most of them are complicated and can only fabricate MNs with specific geometries, which restricts the ability to adjust the performance of the MNs. Herein, we present the fabrication of gelatin methacryloyl (GelMA) MN arrays using the vat photopolymerization 3D printing technique. This technique allows for the fabrication of high-resolution and smooth surface MNs with desired geometries. The existence of methacryloyl groups bonded to the GelMA was verified by ^1^H NMR and FTIR analysis. To examine the effects of varying needle heights (1000, 750, and 500 µm) and exposure times (30, 50, and 70 s) on GelMA MNs, the height, tip radius, and angle of the needles were measured; their morphological and mechanical properties were also characterized. It was observed that as the exposure time increased, the height of the MNs increased; moreover, sharper tips were obtained and tip angles decreased. In addition, GelMA MNs exhibited good mechanical performance with no breakage up to 0.3 mm displacement. These results indicate that 3D printed GelMA MNs have great potential for transdermal delivery of various therapeutics.

## Introduction

Microneedles (MNs) are skin-penetrating painless transdermal drug delivery systems, which consist of sub-millimeter-sized needles (Kim et al., 2012; Mutlu et al., 2021). These devices are considered a minimally invasive medical intervention due to their microscale size (Haq et al., 2009; Krieger et al., 2019). MN arrays can be designed with different sizes and shapes; these devices can play an essential role in the delivery of several therapeutic molecules such as small molecules, biomacromolecules, and nanoparticles for treating various diseases (Larrañeta et al., 2016; Ahmed Saeed AL-Japairai et al., 2020). This next-generation drug delivery system has attracted attention in recent years due to its advantages such as ease of administration, low cost, excellent therapeutic efficacy, and relative safety (Ye et al., 2018; Yang et al., 2019).

Conventional MN fabrication techniques include molding-based techniques (Turner et al., 2021), photolithography (Dardano et al., 2015), micro-milling (Wang et al., 2008), and drawing lithography (Lee and Jung, 2012). However, most of these fabrication techniques can only produce MNs with specific geometries, which limits the capacity to change MN properties such as shape, height, and needle spacing (Donnelly et al., 2010; Donnelly et al., 2011). Furthermore, these techniques are complicated and often require long production processes, manual processing steps, costly equipment, and intensive labor efforts (Wilke et al., 2005; Johnson and Procopio, 2019). Additive manufacturing is a fabrication technique that overcomes many of the design and production limitations associated with conventional MN processing methods (Johnson and Procopio, 2019). 3D printing (more formally referred to as additive manufacturing) approach in which MNs designed in a computer-aided program are produced in a layer by layer manner. 3D printing is also associated with advantages such as high resolution and good cost efficiency (Dabbagh et al., 2021). Digital light processing (DLP) 3D printing technology, which is based on vat photopolymerization, enables the formation of a structure by curing layers of an ultraviolet (UV) sensitive polymer (Kowsari et al., 2018). The projector (digital light micromirror) in the device converts an image signal of the cross-section of the object into a digital signal; the photocuring process takes place using this digital signal (Sun et al., 2021). Using a projector allows for faster print times since it involves curing each full layer of material in one step (Santra et al., 2021). This technique allows only photo-curable materials to be utilized as feedstock materials (Sirbubalo et al., 2021). Moreover, MN production with high resolution on a micrometer scale along with processing of surfaces with smooth features can be performed with this technique (Yang et al., 2021). For example, Shin and Hyun (2021) have demonstrated the fabrication of protein-based MNs using the DLP-based 3D printing technique. In addition, Erkus et al. (2023) prepared GelMA MNs loaded with amoxicillin using DLP 3D printing.

The first-generation materials used in MN fabrication include silicon, metals, ceramics, and glasses (Nikita et al., 2015). These materials have drawbacks in MN manufacturing such as limited drug loading capabilities and expensive production methods (Meng et al., 2020). When compared to other materials, polymeric forms of MNs (soluble and hydrogel-forming MNs) are remarkable due to their unusual properties such as biodegradability, biocompatibility, and an absence of toxicity (Doppalapudi et al., 2014; Azizi Machekposhti et al., 2022). Polymeric hydrogels used as drug delivery systems are defined as three-dimensional network formulations of natural and synthetic polymers (Liu et al., 2020). The network-like porous structure of these structures enhances the loading and controlled release of drugs under proper conditions. It is advantageous to be able to control the drug release profile and performance of hydrogel MNs, which have different degradation profiles and swelling properties (Swain et al., 2021). Gelatin methacryloyl (GelMA), obtained by modifying natural gelatin, is a hydrogel that can be crosslinked using UV light or visible light with exposure to a photoinitiator (Zhao et al., 2016). It is an ideal material for the production of MNs due to its biocompatibility, tunable mechanical properties, printability, low cost, and desirable drug delivery properties (Luo et al., 2019a). In addition, GelMA hydrogel has powerful biological properties such as supporting functional cell growth (Luo et al., 2018). During the GelMA synthesis, many amino groups in the side chains of gelatin are replaced with methacryloyl groups in the structure of methacrylate anhydride (MAA). After synthesis, the methacryloyl groups of gelatin impart crosslinking properties (Pepelanova et al., 2018). The interaction of GelMA with UV light in the presence of a photoinitiator results in the formation of a hydrogel with excellent thermostability (Sun et al., 2018).

When using photopolymers in 3D printing systems, the use of a photoinitiator is necessary to facilitate the crosslinking process (Zhang and Xiao, 2018). During photocrosslinking of photopolymers, the photoinitiator absorbs UV light to generate free radicals; these free radicals polymerize the photosensitive resin to form the polymer network (Zhou et al., 2019). Photoinitiators such as Irgacure 2959, LAP, VA086, and Eosin-Y are commonly used owing to their cytocompatibility with living cells (Nikita et al., 2015). In particular, LAP is a remarkable photoinitiator for biomedical applications due to its water solubility, low toxicity, and absorbance of both 365 and 405 nm light (Mau et al., 2019).

Herein, GelMA MN arrays with the desired design at different exposure times were developed using the DLP-based 3D printing technique. As far as is known, no previous studies have been reported in the literature regarding DLP-based GelMA MN arrays fabricated at different heights and exposure times. In the current study, after the GelMA was synthesized, the design, printing conditions, and post-printing processes of MNs were optimized. Mechanically and morphologically optimized GelMA MNs may represent an attractive component in new types of transdermal drug delivery systems. The findings of this study can offer insight into design applications aimed at optimizing 3D-printed MNs for adjustable and customizable drug delivery.

## Materials and methods

### Materials

Gelatin Type A obtained from porcine skin, methacrylic anhydride (MAA), lithium phenyl-2,4,6-trimethyl-benzoyl phosphinate (LAP), and dialysis membrane (with a cut-off value of 14 kDa and an average flat width of 43 mm) were purchased from Sigma-Aldrich (Darmstadt, Germany). Sodium carbonate, sodium hydroxide, and hydrochloric acid fuming 37% were obtained from Merck KGaA (Darmstadt, Germany). Sodium hydrogen carbonate (>99.7%) was obtained from ISOLAB (Eschau, Germany). Phosphate-buffered saline (PBS, pH 7.4) was purchased from ChemBio (Turkey).

### Synthesis of gelatin methacryloyl (GelMA)

**FIGURE 1:**
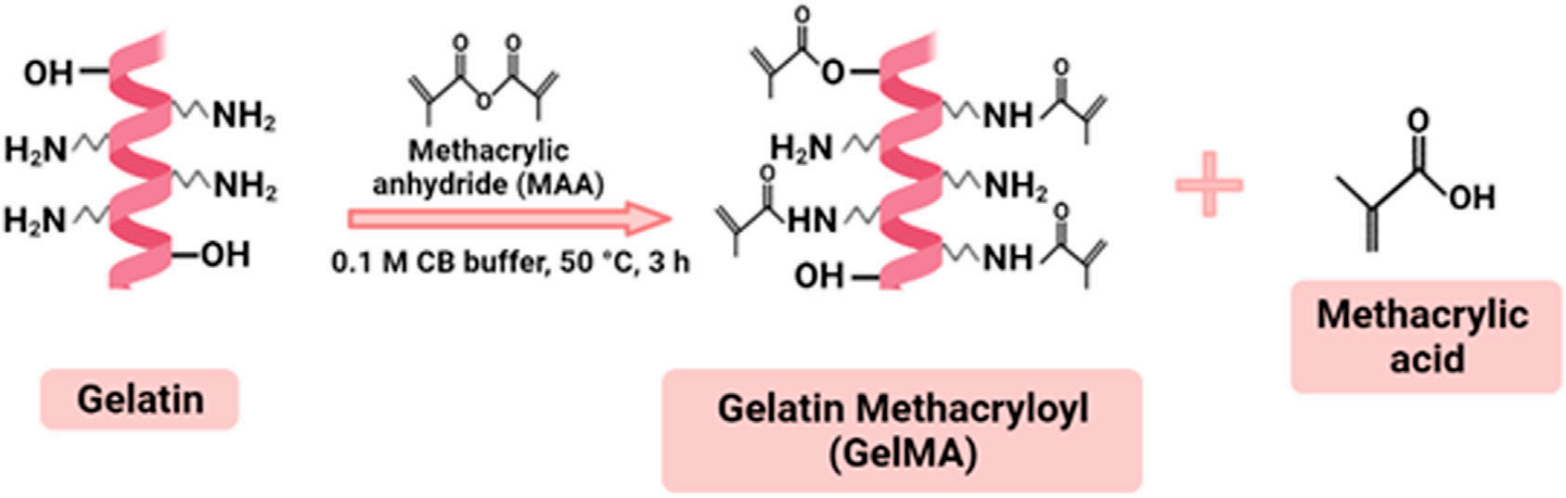
Synthesis route of GelMA.

10% (w/v) solution of type A gelatin was prepared in 0.1 M carbonate bicarbonate buffer (0.1 M CB buffer containing 3.18 g sodium carbonate and 5.86 g sodium bicarbonate in 1 L of distilled water, pH 9) at 60°C. Then, 0.1 ml of methacrylic anhydride (MAA) per Gram of gelatin was added to the gelatin solution and allowed to react for 3 h at 50°C under constant stirring. The reaction was then terminated by adjusting the pH to 7.4 (Figure 1). The obtained solution was dialyzed with a 14 kDa molecular-weight-cutoff (MWCO) membrane against distilled water for 2 days at 40°C. Dialysis of GelMA solution helped to remove unreacted MAA and methacrylic acid byproducts. After the dialysis step, the solution was lyophilized for 3 days and stored at +4°C until use.

### Determination of GelMA degree of substitution

The degree of substitution (DS) of GelMA was investigated using ^1^H NMR spectroscopy (Bruker Avance III 600 MHz, Bremen, Germany). Both gelatin and GelMA were dissolved at a 10 mg/ml concentration in D_2_O; ^1^H NMR spectra were obtained at a frequency of 600 MHz and at room temperature. The DS of GelMA was calculated according to the following equation:

$$DS\left(\%\right)=\left(1-\frac{peak\;area\;of\;GelMA\;lysine\;methylene}{peak\;area\;of\;gelatin\;lysine\;methylene}\right)x100$$ (1)

### Fourier transform infrared spectroscopy (FTIR)

The chemical structure of gelatin and GelMA were investigated using Fourier transform infrared spectroscopy (FTIR, FT/IR-ATR 4700, Jasco, Easton, MD, USA) at room temperature. Spectra were obtained between 450 and 4000 cm^−1^ range at a resolution of 4 cm^−1^.

### Rheological characterization of GelMA

The rheological behavior of GelMA hydrogel was analyzed using a digital rheometer (Discovery HR2, TA Instruments, New Castle, DE, United States). The oscillation mode was selected to determine the temperature dependence of the shear modulus of the GelMA hydrogel. The temperature ramp test was performed over a range of 37°C–15°C (heating rate 1°C/min) with a frequency of 1 Hz and strain of 1%. The frequency sweep test was carried out at an angular frequency of 0.01–100 rad/s with a constant frequency (1 Hz) and strain (1%). The viscosity of GelMA hydrogel was measured by varying the shear rate from 1 to 100 1/s.

### Design and fabrication of GelMA MNs

**FIGURE 2:**
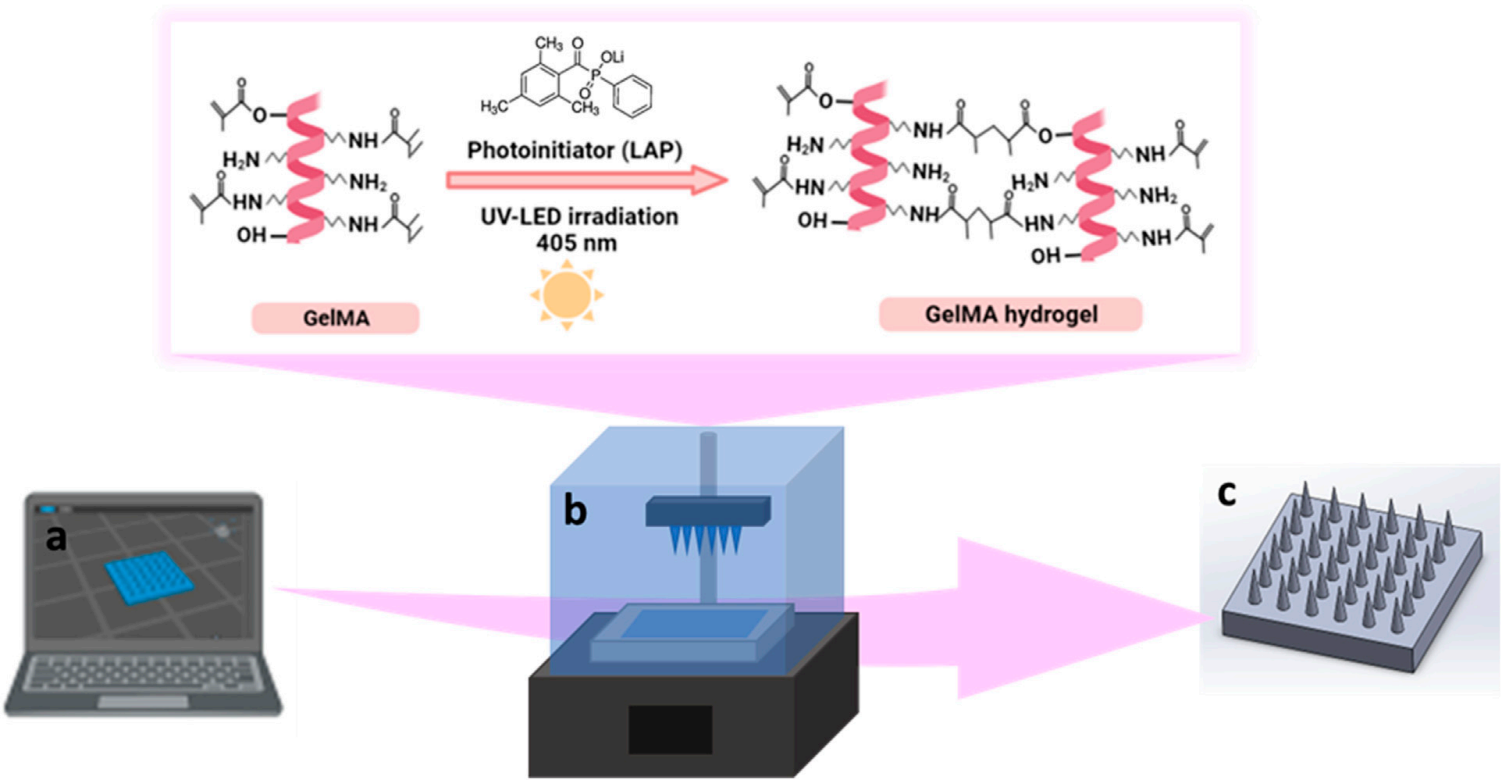
Schematic representation of the design and fabrication of GelMA MNs, **(A)** designing of CAD model, **(B)** photocrosslinking of GelMA hydrogel during DLP printing, **(C)** 3D printed GelMA MNs.

Computer-aided design (CAD) files of conical MN arrays with three different needle heights of 1000, 750, and 500 µm were prepared using SolidWorks 2020 (Dassault Systèmes SE, Vélizy-Villacoublay, France). MNs were designed to be 600 µm wide at the base and were attached to a solid 10 × 10 × 1 mm substrate consisting of a 6 × 6 array. The MN designs were converted to the stl file format and sliced using Chitubox (Shenzhen Chuangbide Technology Co., Ltd., Shenzhen City, China), the software for the 3D printer (Figure 2A). A commercially accessible DLP-based 3D printer (Phrozen Shuffle 4K, Phrozen Tech Co Ltd., Hsinchu, Taiwan) was utilized to manufacture the MNs. A 12 mW/cm^2^ light intensity and 405 nm light wavelength were used for printing the MNs. Subsequently, MNs with varying heights were subjected to exposure times of 30, 50, and 70 s, respectively.

For the printing of MNs, a 10% (w/v) GelMA solution was prepared with PBS solution under constant stirring at 40°C for 30 min. The photoinitiator (LAP) at a 0.5% concentration was added to the GelMA solution and mixed for 10 min. Next, the mixture was brought to room temperature (∼25°C) and transferred to the tank of the DLP printer (Figure 2B). MNs were printed according to the parameters given above. The fabricated 3D-printed MNs were dried in the dark at room temperature for 24 h and kept in a dehumidified container until the characterization activities (Figure 2C).

### Morphological characterization of GelMA MNs

The height, tip radius, angle, and surface characterization of each MN were evaluated using a scanning electron microscope (SEM) (EVA MA 10, Zeiss, Jena, Germany). Prior to analysis, the surfaces of the MNs were coated with gold using a spray coating machine (SC7620, Quorum, Laughton, East Sussex, UK) for 120 s.

### Mechanical analysis for GelMA hydrogels and GelMA MNs

10% GelMA hydrogels prepared with different exposure times (30, 50, and 70 s) were characterized in terms of their compression stiffness using a compression testing machine (EZ-LX, Shimadzu, Kyoto, Japan). Cylindrical specimens of GelMA (8 mm in diameter and 6 mm in height) were tested; a rate of 1 mm/min and a maximum strain of 60% were used in these studies. Compressive modulus values were calculated from the initial linear region (0%–20% of strain) of the obtained stress-strain curves. Each measurement was performed in triplicate and results are reported as mean ± standard deviation values.

The mechanical strength of GelMA MNs was analyzed with a compression testing machine (EZ-LX, Shimadzu, Kyoto, Japan). MNs were placed on a stainless steel plate at a distance of 2 mm; an axial force was applied at a constant rate of 0.1 mm/min perpendicular to the axis of the MNs. The mechanical characteristics of MNs with different needle heights (1000 μm, 750 μm, and 500 µm) and different exposure times (30, 50, and 70 s) were profiled. All tests were performed in triplicate.

### Statistical analysis

The experiments were carried out at least in triplicate, and data are expressed as mean ± standard deviation (SD). Post-hoc one-way ANOVA with a Tukey-Kramer pair-wise comparison were employed for statistical analysis. A value of *p* ≤ 0.05 is considered statistically significant, and additional significance is indicated by ** for *p* < 0.01 and *** for *p* < 0.001.

## Results and discussion

### Determination of GelMA degree of substitution

**FIGURE 3:**
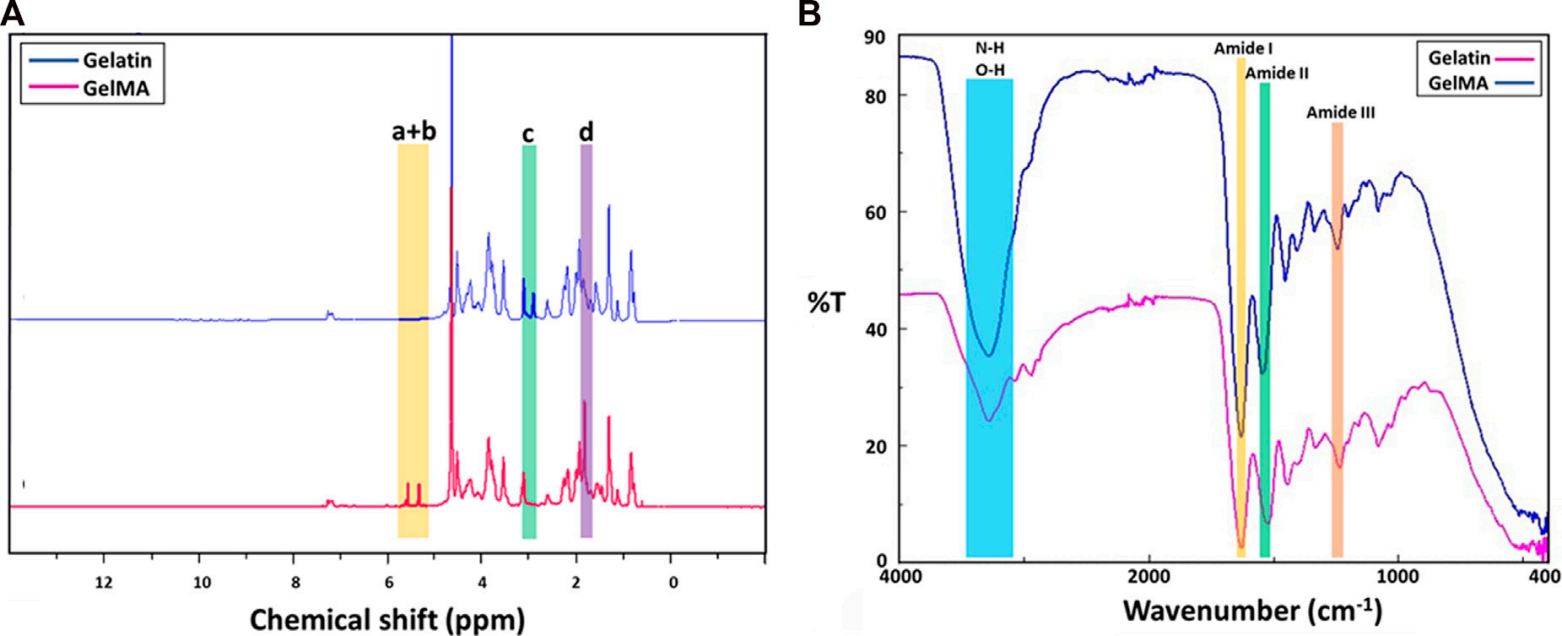
^1^H NMR spectra of gelatin and GelMA **(A)**, FTIR spectra of gelatin and GelMA **(B)**.

^1^H NMR analysis was performed to verify the successful substitution of gelatin with methacryloyl groups (Figure 3A). Compared with the ^1^H NMR spectra of gelatin, the GelMA displayed new signals corresponding to the methacryloyl groups, labelled as orange (a+b), green (c) and purple (d). The signals at around chemical shifts of 5.3 and 5.6 ppm (a+b) were attributed to the acrylic protons (2H) of methacryloyl group grafted to lysine and hydroxylysine residues of the gelatin backbone. This result indicates the existence of C=C bonding in the anhydride structure, which is related to presence of the vinyl groups of methacrylate anhydride (Farasatkia et al., 2021). The signal at approximately 1.8 ppm (d) in the GelMA spectrum was assigned to the methyl protons (3H) of the grafted methacryloyl group. In addition, a decrease in the intensity of the signal at around 2.9 ppm (c), which was associated with the lysine methylene (2H) was observed in GelMA compared to gelatin (Raveendran et al., 2019; Zu et al., 2021). As lysine is the reaction site, this finding was used to quantify the DS, which was estimated to be 75.4%.

### Fourier transform infrared spectroscopy (FTIR)

The substitution of methacrylate groups to the gelatin chain in the structure of GelMA was further confirmed by FTIR analysis. The FTIR spectra of pure gelatin and GelMA are presented in Figure 3B. The FTIR spectrum of GelMA exhibits a sharp peak at 1630 cm^−1^, corresponding to C=O stretching groups of amide I bond (labelled as yellow) (Farasatkia et al., 2021). The peak at 1546 cm^−1^ is attributed to the N–H bending groups of the amide II bond (labelled as green); the peak at 1244 cm^−1^ is related to the C–N stretching and N-H bending of the amide III bond (labelled as pink) (Xiang et al., 2020; Farasatkia et al., 2021). Furthermore, the peak located in the range of 3200–3400 cm^−1^ (labelled as blue) detected in GelMA is associated with the existence of peptide bonds (N–H stretching) and -OH functional groups (Sreekumaran et al., 2021). The shifts and changes identified in the GelMA peaks compared to pure gelatin indicated that the lysine groups of gelatin were successfully substituted by the methacrylate groups (Rahali et al., 2017).

### Rheological characterization of GelMA

**FIGURE 4:**
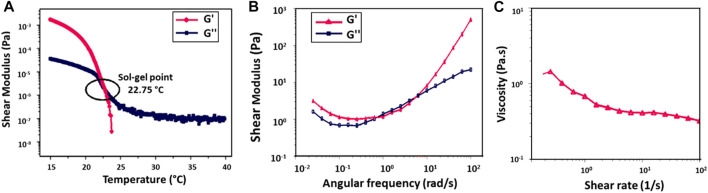
Rheological analysis of 10% (w/v) GelMA hydrogel, **(A)** temperature ramp test, **(B)** frequency sweep test, and **(C)** rotational shear rate-viscosity measurement.

To determine the viscoelastic properties of 10% (w/v) GelMA hydrogel, its shear moduli and viscosity were evaluated by rheological testing (Figure 4). The hydrogel demonstrated temperature-dependent gelation behavior (Figure 4A). The crossing of the storage modulus (G′) and loss modulus (G″) curves is the gelation temperature of the hydrogel. Below about 22°C, the GelMA hydrogel exhibited solid characteristics (G′ > G″). When the temperature was increased, the sol-gel transition occurred in the range of 22°C (G′ = G″); at higher temperatures, the hydrogel displayed liquid-like behavior (G'' > G′). According to the results of the oscillation frequency sweep of the GelMA hydrogel tested in the angular frequency range of 0.01 rad/s to 100 rad/s, an increase was observed in both G′ and G″ with increasing frequency (Figure 4B). In Figure 4C, the viscosity decreased with increasing shear rate, supporting the shear-thinning behavior of the GelMA hydrogel (Luo et al., 2020).

### Morphological characterization of GelMA MNs

**FIGURE 5:**
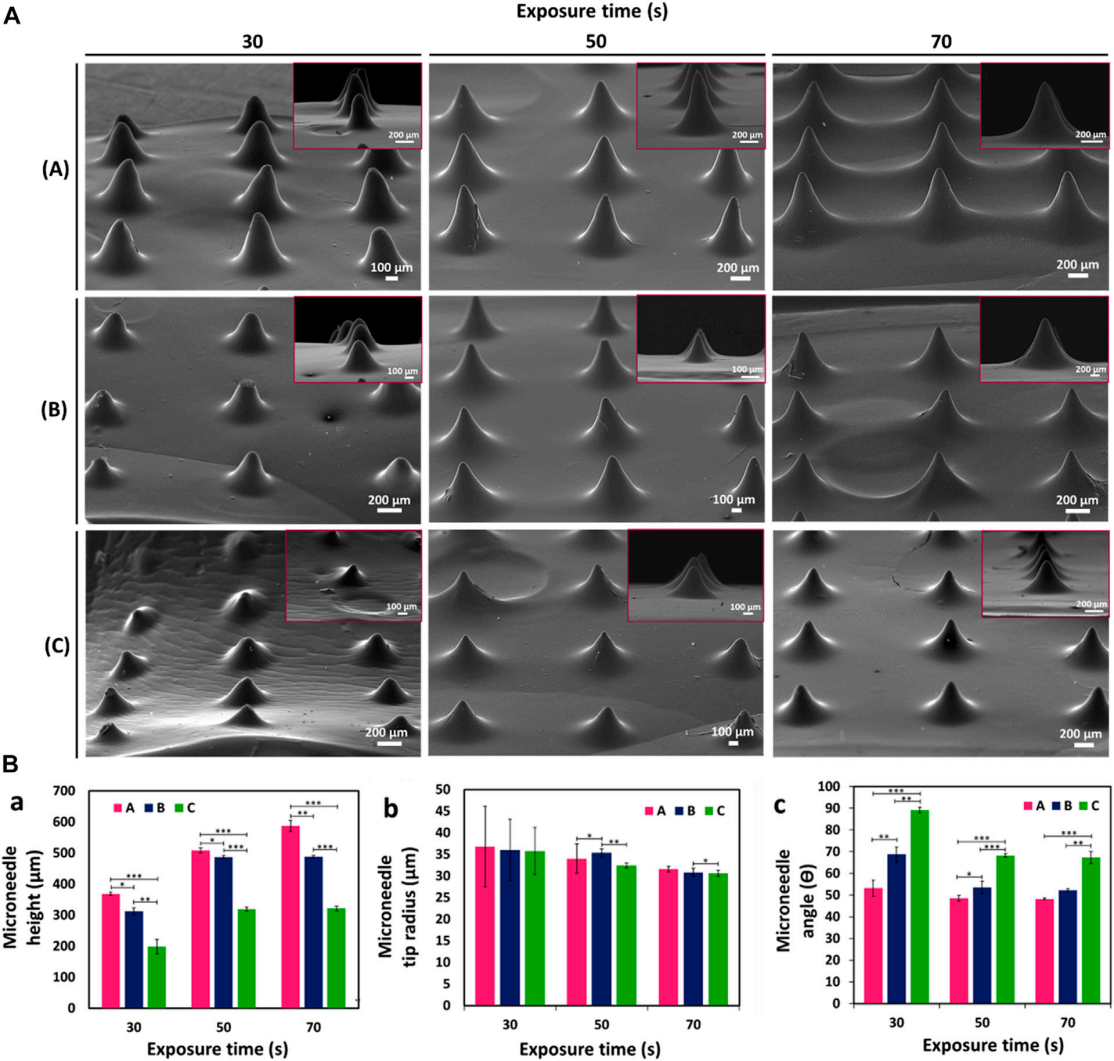
**(A)** SEM images of GelMA MNs designed in three different needle heights according to varying exposure times: (a) 1000 µm height, (b) 750 µm height, and (c) 500 µm height. **(B)** Variation of (a) height (b) tip radius and (c) angle of GelMA MNs according to exposure times. Statistical difference is indicated with *p ≤ 0.05, **p < 0.01, and ***p < 0.001. Error bars represent the standard deviations (SDs) of measurements performed on at least three samples.

GelMA MNs designed with three different needle heights (h:1000, 750, and 500 µm) were fabricated with a DLP printer by utilizing three different exposure times (30, 50, and 70 s); SEM images of the obtained hydrogel MNs are shown in Figure 5. The effect of varying needle sizes and exposure times on GelMA MNs was investigated. As can be seen in Figure 5A, MNs with an exposure time of 30 s could not be fully printed as the light intensity may drop below the threshold as the sliced images approached the tip (Yao et al., 2020), preventing the processing of the conical structure. Incomplete MNs resulting from a short exposure time are not hard enough to penetrate the skin (Gao et al., 2018). On the other hand, MNs exposed for 50 s were well-printed conical structures that exhibited a uniform and regular morphology. When the exposure time was increased to 70 s, the distance between the MNs decreased due to overexposure. In addition, the hardness of the MNs may increase as a result of the long exposure time (Luo et al., 2019b).

In order to better observe the influences of changing needle sizes and exposure times on the printability of MNs, three parameters (e.g., height, tip radius, and angle) of the printed MNs were measured and presented in Figure 5B. The printability of MNs at different heights is important for adjusting the depth of penetration in the skin and changing the volume available for the delivery of therapeutics (Yan et al., 2010). It is seen that the experimental heights of MNs, which are designed with theoretical needle heights of 1000 µm (A), 750 µm (B), and 500 µm (C), respectively, are less than those of the corresponding designs (Figures 5B,a). This finding may be due to the minimum UV dose necessary for photopolymerization as well as the manner in which a layer is produced (Krieger et al., 2019). The light that projects off each micromirror usually spreads to nearby pixels. Thus, the amount of light per unit area for large pieces (where light from surrounding pixels converge) is greater than those for small pieces. Therefore, the curing of small pieces is often not achieved (Johnson and Procopio, 2019). Accordingly, since a vertically aligned needle exhibits a continuously decreasing cross-sectional slice in the x-y plane, the print may result in round-shaped tips before the full theoretical needle height is reached (Krieger et al., 2019). Similar results were noted by Johnson et al. (2016) in a study that involved the CLIP (Continuous Liquid Interface Production) printing system. In addition, the heights of the MNs increased as the exposure time increased (from 30 to 70 s) for all three needle sizes as confirmed by SEM images: (A) MNs increased from about 368.4 ± 5.6 µm to 586.9 ± 17.9 µm, (B) MNs increased from 312.2 ± 10.8 µm to 488.1 ± 2.9 µm, and (C) MNs increased from 197.8 ± 23.5 µm to 321.1 ± 7.4 µm.

Another important parameter, that is, necessary to ensure penetration of the MNs into the skin is the tip radius. The tip radius determines the sharpness of the MN (Johnson and Procopio, 2019). Figures 5B,b demonstrates that the tip radii of the printed MNs were in the range from ∼30 μm to 36 μm. There appeared to be a tendency to decrease in tip radius with decreasing needle height (from 1000 to 500 µm) at the same exposure time. When measurement precision and error are taken into consideration, the difference is not substantial. Needle tips are particularly subject to needle-to-needle variability as a result of a lack of precision in printing (Krieger et al., 2019). Moreover, as expected, sharper tips were obtained as the exposure time increased for all three needle sizes. (C) MNs exhibited the sharpest needle tip with a tip radius of 30.6 ± 0.7 at 70 s. Since tip radii of 20–40 µm are known to be of sufficient sharpness for skin penetration (Lee et al., 2008), the obtained tip radii can be said to be among the sharpest hydrogel needles printed using a DLP printing system.

The θ angle was the last design parameter measured. Regardless of the tip radius, this value measures the angle between needle sides. According to Figures 5B,c, the needle angle increased as needle height decreased for the same exposure time. As exposure time increased, the needle angle decreased for (A) MNs from 53.2 ± 3.7 to 48.2 ± 0.4, (B) MNs from 68.7 ± 3.5 to 52.2 ± 0.6, and (C) MNs 89.1 ± 1.2 to 67.3 ± 2.6, respectively. These results support the successful fabrication of GelMA MNs with different needle heights and exposure times using a DLP printer.

### Mechanical analysis for GelMA hydrogels and GelMA MNs

**FIGURE 6:**
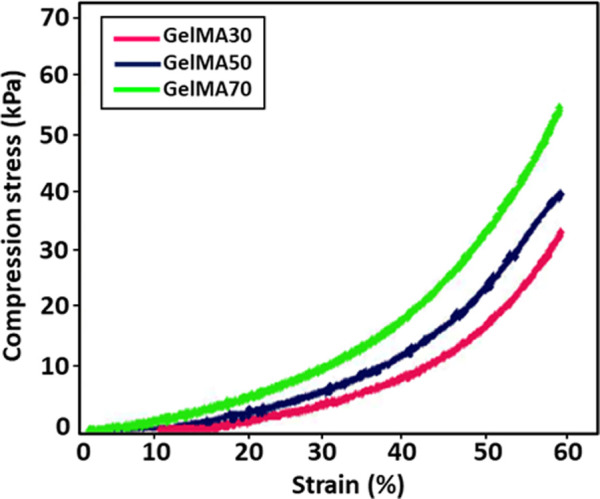
Stress-strain curves for GelMA hydrogels at different UV exposure times (30, 50, and 70 s).

Figure 6 shows the compressive stress-strain curves of GelMA hydrogels at different UV exposure times (30, 50, and 70 s). From the stress-strain curves, it was observed that the compressive strength increased by increasing the exposure time from 30 to 70 s. As can be seen in Table 1, the GelMA hydrogel exposed to 70 s exhibits a higher modulus of compression than those exposed to 30 and 50 s. Since polymerization is a kinetic process, longer exposure times enable greater completion of the polymerization reaction. Thus, longer polymerization times up to a certain threshold lead to a greater number of functional crosslinks, resulting in higher modulus of elasticity values (Sheth et al., 2017). Similar results for the GelMA hydrogel have been reported by Chansoria et al. (2021).

**TABLE 1:** Compressive modulus of elasticity from stress-strain curves for GelMA hydrogels at different UV exposure times.

Sample		Compressive modulus (kPa)	
10% (w/v) GelMA	30 s	50 s	70 s
	1.97 ± 0.12	3.72 ± 0.10	6.51 ± 0.10

**FIGURE 7:**
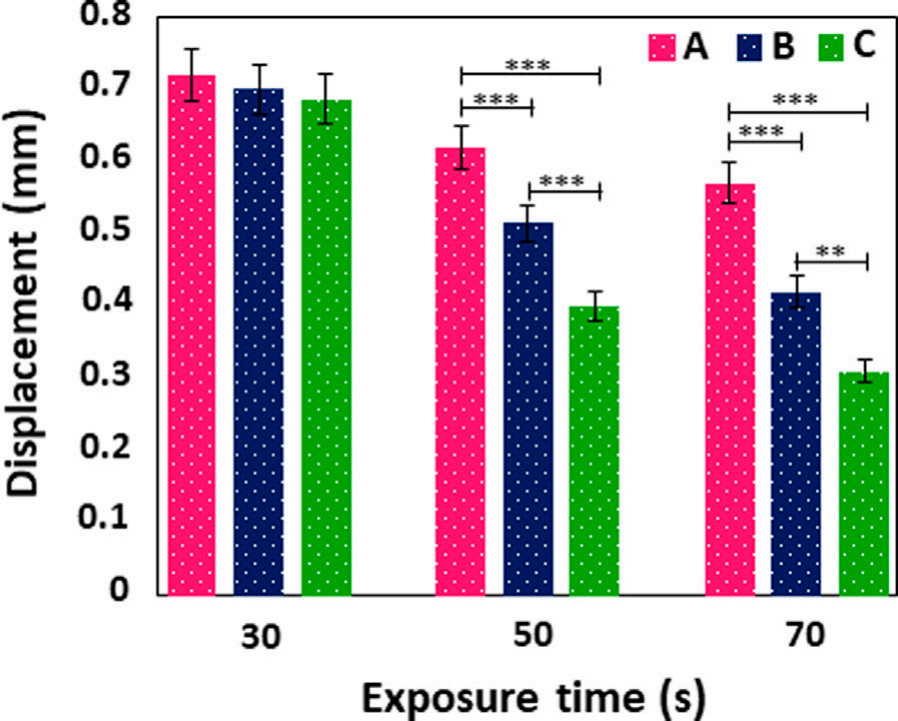
Displacement curve of GelMA MNs with different needle heights according to varying exposure times. Statistical difference is indicated with *p ≤ 0.05, **p < 0.01, and ***p < 0.001. Error bars represent the standard deviations (SDs) of measurements performed on at least three samples.

The mechanical properties of MNs are critical for successful skin penetration (Li et al., 2019). The mechanical properties of GelMA MNs was investigated using a compression test. As shown in Figure 7, no discontinuity or breakage was observed in the displacement curves of the GelMA MNs up to the displacement value of 0.3 mm. In addition, there was no broken MN up to the value above. Makvandi et al. (2021) noted that transdermal MN patches should puncture the human stratum corneum (∼10–20 μm) without tearing or bending during penetration. This result implies that the produced MNs exhibit appropriate toughness for skin penetration (Erkus et al., 2023). Moreover, an increase in mechanical strength with an increase of exposure time (from 30 to 70 s) for the same needle size groups was one of the findings supported by previous studies (Yao et al., 2020; Economidou et al., 2021). The amount of force required to induce the same level of compression in materials with increased crosslinking density was higher, indicating that increasing the crosslinking time improved the mechanical strength of MNs significantly. Therefore, the quantity of crosslinking in the GelMA MNs is a key factor in determining the mechanical qualities of MNs (Luo et al., 2019b). By profiling the applied compressive force and the displacement of the MNs, Zhou et al. found that longer crosslinking times resulted in higher crosslinked network densities, which required greater force to achieve similar displacement values (Zhou et al., 2020). In another study, when the difference in the mechanical properties of the produced GelMA MNs was examined without any UV crosslinking and after 15 s of crosslinking, it was demonstrated that the mechanical strength of the 15 s light-cured GelMA MNs increased significantly (Zhao et al., 2021).

Moreover, Figure 7 demonstrates the displacement of MNs at different heights exposed to UV against an applied force. It was determined that the longest MNs in each group, among the MNs produced in three different sizes, tended to move the most. It can be observed that the displacement values of (A) MNs were high among their groups in those exposed to 30 s of UV, (A) in those exposed to 50 s of UV, and (A) in those exposed to 70 s. It should be noted the MNs produced by Xenikakis et al. with a maximum length of 930 μm at applied forces over 60 N were severely bent and deteriorated compared to short needles (Xenikakis et al., 2019). In a different study, MNs produced with the same base diameter of 750 and 500 µm had their displacements measured as 0.0255 and 0.127 mm, respectively, when 0.1 N force was applied. This result proves that higher MNs exhibited more displacement under the same force (Gittard et al., 2013).

## Conclusion

In this study, GelMA MNs were successfully fabricated with desired geometries at high resolution using the DLP-based 3D printing technique. Smooth surface MNs with different heights (1000, 750 and 500 μm) and different UV exposure times (30, 50, and 70 s) were obtained. ^1^H NMR analysis proved the existence of methacryloyl groups attached to GelMA; these results were also supported by FTIR analysis. According to the morphological analysis, it was observed that MNs had higher needle heights and sharper tips as the exposure time increased; the tip angles decreased accordingly. Furthermore, the compression test results showed that increasing the exposure time decreased the amount of displacement. Therefore, it can be said that GelMA MNs at lower heights showed less displacement when equal force was applied. In addition, GelMA MNs demonstrated good mechanical performance without any breakage up to 0.3 mm displacement. These findings indicate that the 3D-printed GelMA MNs have the potential for use in a variety of transdermal drug delivery systems.

## Data Availability

The raw data supporting the conclusion of this article will be made available by the authors, without undue reservation.
